# A peptidic hydrogel that may behave as a “Trojan Horse”

**DOI:** 10.3762/bjoc.9.44

**Published:** 2013-02-22

**Authors:** Nicola Castellucci, Giorgio Sartor, Natalia Calonghi, Carola Parolin, Giuseppe Falini, Claudia Tomasini

**Affiliations:** 1 Dipartimento di Chimica “Ciamician”, Università di Bologna, Via Selmi 2, I–40126 Bologna, Italy; 2 Dipartimento di Farmacia e Biotecnologie, Università di Bologna, Via Irnerio 48, 40126, Bologna, Italy

**Keywords:** amino acids, confocal microscopy, controlled release, hydrogel, low molecular weight hydrogelator

## Abstract

A physical hydrogel prepared with the low-molecular-weight hydrogelator (LMWHG) CH_2_(C_3_H_6_CO-L-Phe-D-Oxd-OH)_2_ and water/ethanol mixture was applied as a potential “Trojan Horse” carrier into cells. By SEM and XRD analysis we could demonstrate that a fibrous structure is present in the xerogel, making a complex network. The gelator is derived from α-amino acids (Thr, Phe) and a fatty acid (azelaic acid) and is biocompatible: it was dosed to IGROV-1 cells, which internalized it, without significantly affecting the cell proliferation. To check the internalization process by confocal microscopy, fluorescent hydrogels were prepared, introducing the fluorescent dansyl moiety into the mixture.

## Introduction

Drug delivery is an important topic in the finding of successful drugs, which should be sufficiently polar for ease of administration and successful distribution in the organism, but also adequately hydrophobic so as to traverse the lipid bilayer of the cell membrane. Because they do not fulfill these physical properties, a large number of drug leads fail to make it to clinical trials, and they are, thus, often extensively modified to enhance their solubility in water [1]. A different approach consists of using a vector able to be internalized by cells and then loading the drug molecule on to it as cargo [2]. A wide family of molecules can be used to exploit this “Trojan Horse” strategy. Many peptides can penetrate mammalian cell membranes and take cargo with them. To enable penetration, initial binding of the peptide to the cell surface through electrostatic interactions with lipids, presumably followed by membrane destabilization to allow translocation, must occur [3]. The general mechanism of this uptake remains elusive and appears to be unrelated to the presence of specific receptors on the membrane surface or even receptor binding [4]. The peptide secondary structure is very important, as usually the α-helix conformation interacts more easily with the cell membrane surface than does the β-sheet conformation [5].

Low-molecular-weight hydrogelators (LMWHGs) are defined as small molecules that self-assemble into long fibers, resulting in the formation of a gel [6–10]. Recently, several examples of peptide or peptidomimetic gelators have been reported [11]. In the gels, noncovalent, weak interactions between the individual LMWHG molecules hold the fibers together. The gel-forming characteristics of LMWHGs can be controlled by altering the strength of these interactions, as illustrated by the thermal reversibility of the gel–sol transition. They have a wide range of applications in biomaterials, biosensors, tissue engineering, and drug delivery [12–15]. The interest in these materials as tools for the controlled release of drugs is mainly linked to their capability to release gel-entrapped molecules in response to external stimuli, such as changes in pH, ionic strength and temperature [16–18].

## Results and Discussion

We have recently described the synthesis and the application of a small library of stereoisomeric compounds made of azelaic acid (a long chain dicarboxylic acid) coupled with different pseudopeptides, all containing the L-Phe-D-Oxd (Oxd = 4-carboxy-5-methyloxazolidin-2-one) moiety [19–21]. This skeleton is the minimal framework of a class of foldamers, having the ability to self-organize. We have also shown that stable LMWGs are formed by stoichiometric mixtures of pseudopeptides and metal ions. The potential use of these materials as vectors to carry drug molecules has been proposed [22].

**Figure 1:**
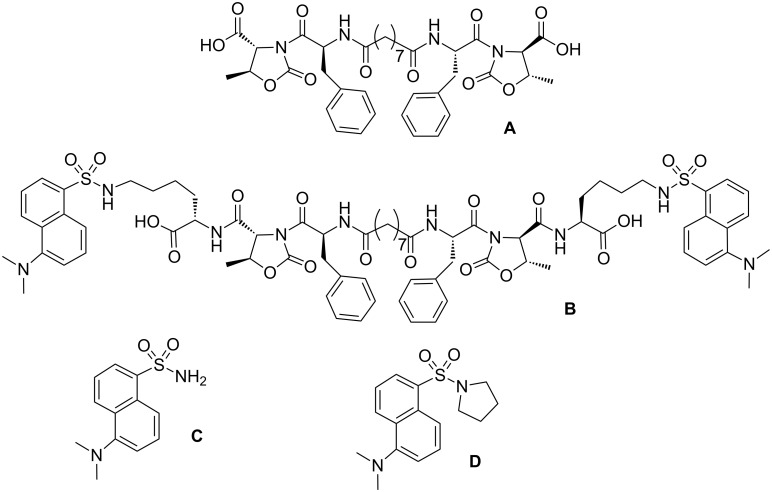
Chemical structure of the LMWHGs **A** and **B** and of the fluorescent moieties **C** and **D** described in this work.

The aim of this work is the preparation of hydrogels that readily cross the membrane barrier of the ovarian cancer cell line IGROV-1 and are well tolerated by them, so that they may behave as a “Trojan Horse” and carry drug molecules into the cell. For this purpose, we used the gelator **A** (Figure 1), derived from natural proteinogenic amino acids (Thr, Phe) and a fatty acid (azelaic acid). Compound **A** was prepared from Boc-L-Phe-D-Oxd-OBn and azelaic acid, as previously reported [23]. To check the cellular uptake by confocal laser scanning microscopy, we prepared also three fluorescent compounds **B**, **C** and **D**, all containing the fluorescent dansyl group (Dans) [24].

First we prepared hydrogels **1** and **2** (Table 1), both containing **A** and a mixture of water and ethanol in 9:1 ratio and 1:1 ratio, respectively.

**Table 1:** Gelling status of the molecules **A**, **B**, **C** and **D** and of their mixtures.

hydrogel	LMWHG	weight (mg)	solvent (H_2_O/EtOH)	outcome

**1**	**A**	3.7	9:1	**gel**
**2**	**A**	3.7	1:1	**gel**
**3**	**B**	3.7	9:1	sol.
**4**	**B**	3.7	1:1	sol.
**5**	**B**	7.3	9:1	sol.
**6**	**B**	7.3	1:1	**gel**
**7**	**A** **B**	3.7 0.4	9:1	**gel**
**8**	**A** **C**	3.7 0.4	9:1	**gel**
**9**	**A** **D**	3.7 0.4	9:1	**gel**

Both samples easily form gels, as shown in Table 1. They were prepared, by dissolving the gelator in a test tube (8 mm wide) containing ethanol. The mixture was shaken for a few minutes by hand, then water was added and the tube was sonicated for fifteen minutes at room temperature and left to stand for an additional 16 hours for the gel formation. Interestingly, these LMWHG having hydrophilic carboxylic acid moieties, located in the hydrophilic part of the molecule, provide a reversible response to changes in pH. Indeed they are in the gel state up to pH ≈ 9. At higher pH, the gels undergo conversion to the sol status, due to the almost complete ionization of the carboxylate groups [25].

Then, following the same method, we attempted to prepare hydrogels containing the fluorescent dansyl group (Dans).

First we tried with pure **B** (Table 1, entries 3–6), but we could verify that **B** is not as good a gelator as **A**. Indeed only **6** readily forms a gel: it requires a higher concentration of gelator (0.74% w/v compared to 1,2 which require only 3.7% w/v of **A**) and a 1:1 water/ethanol mixture, probably because **B** is less hydrophilic than **A** [26]. Thus, we followed a different approach, preparing hydrogels **7–9** with **A** as gelator, doped by a small amount of the dansyl-containing compounds **B**, **C** and **D**. These all readily form fluorescent hydrogels, which may be used to evaluate the cellular uptake of **A**.

Before evaluating the cellular uptake of the hydrogels, the structure of **1** (hydrogel), and its corresponding xerogel, were analyzed by X-ray diffraction (XRD) and scanning electron microscopy (SEM). Hydrogel **1** is a strong and thermoreversible gel (Figure 1a) with its melting point at 45 °C.

**Figure 2:**
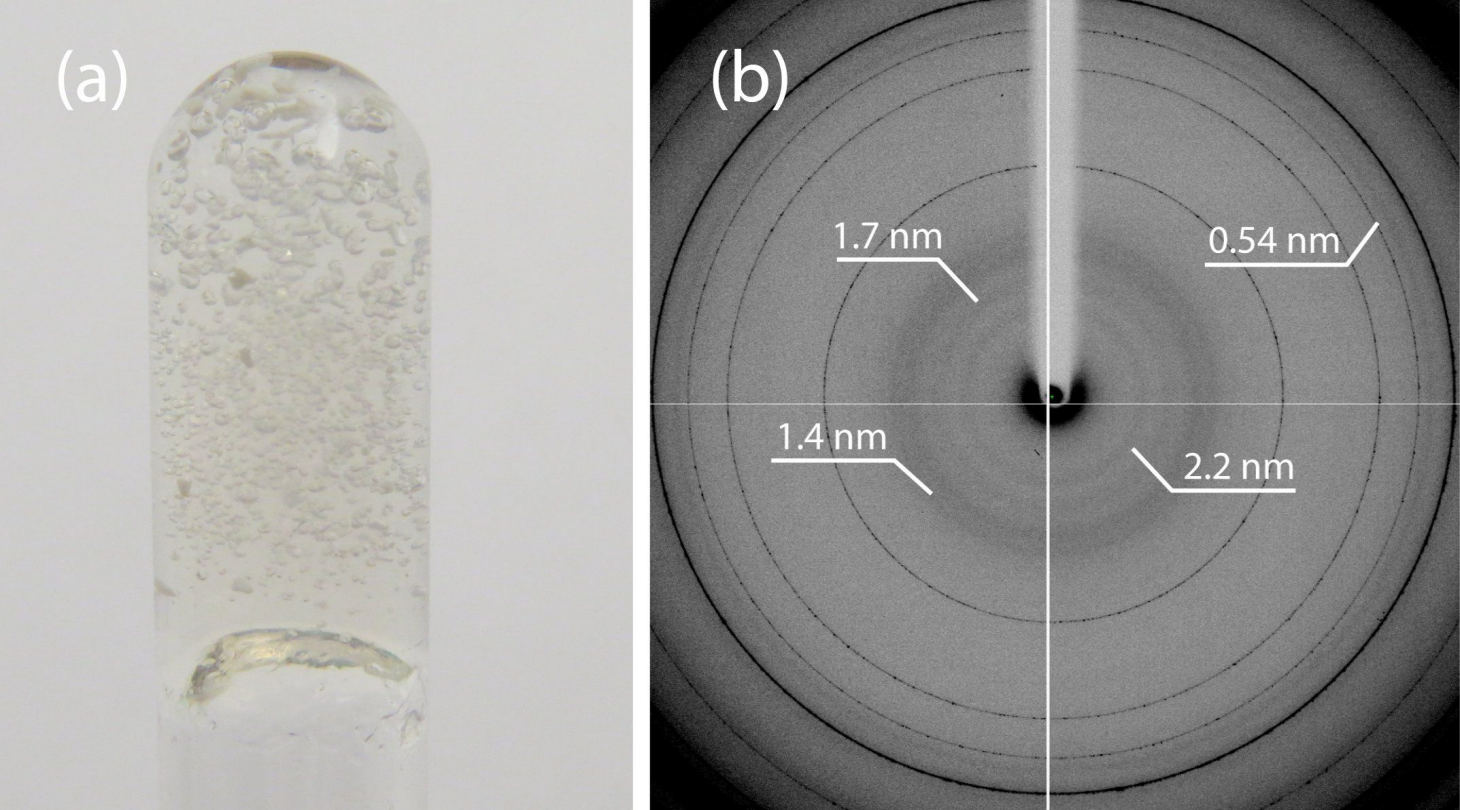
(a) Camera picture of hydrogel **1** in an upside down glass test tube and (b) low–medium-angle XRD pattern from a fragment of the hydrogel **1** collected at 100 K. In (b) the periodicities associated with diffraction rings due to the ordered assembly of **1** are indicated. Those not listed are due to ice.

The XRD pattern from the hydrogel **1**, collected at 100 K, is characterized by the presence of diffraction rings at low angle (indicated in Figure 2b) having periodicities of 2.2 nm, 1.7 nm, 1.4 nm and 0.54 nm. The additional high-angle diffraction rings not listed are due to the presence of ice [27]. Any attempt to collect XRD data at room temperature, or at 100 K in the presence of cryoprotectants, to avoid the formation of ice, failed to show the low-angle diffraction effects.

**Figure 3:**
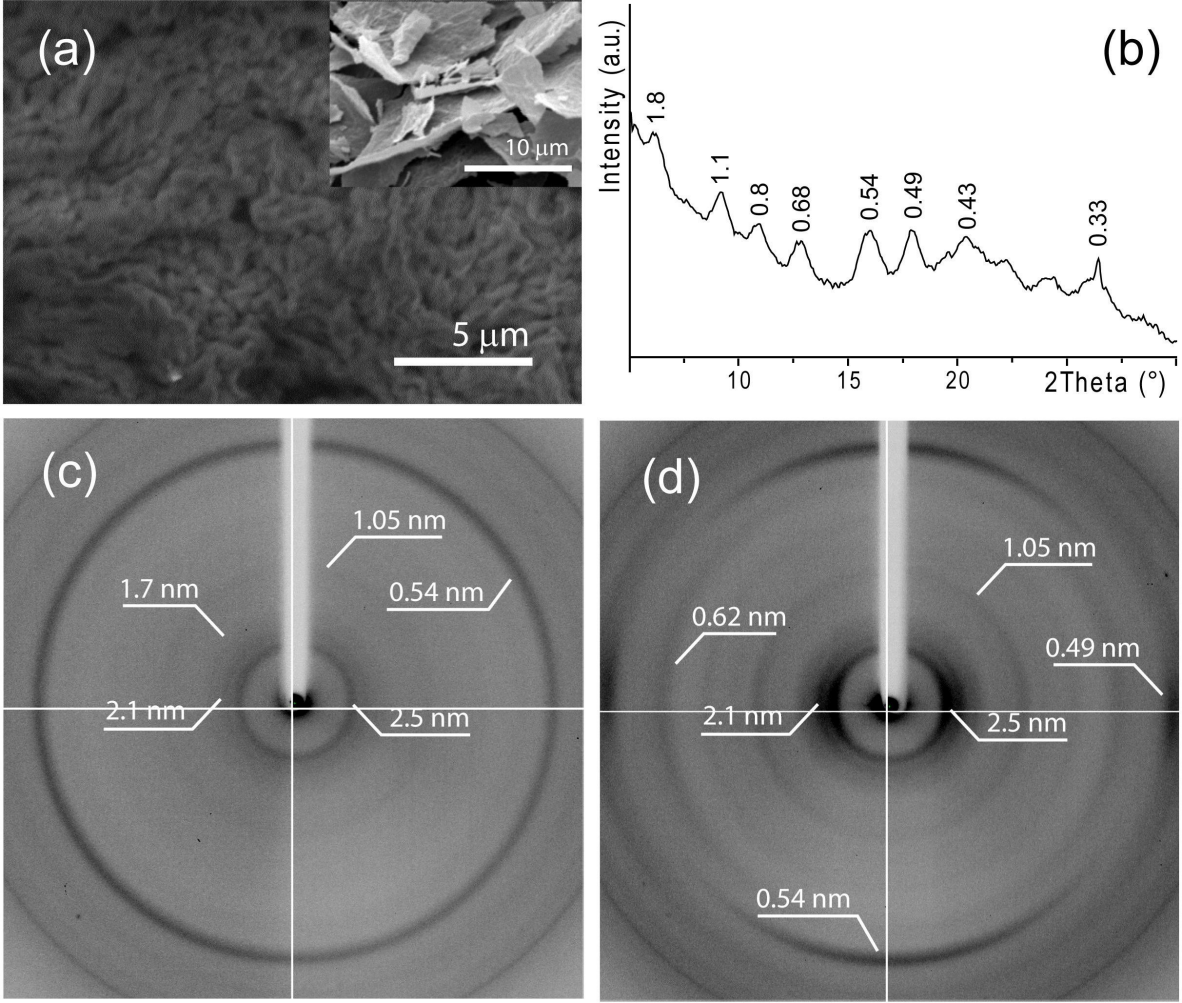
(a) Scanning electron microscopy images of a xerogel **1** film fragment. In the inset a low magnification view of the xerogel film fragments is shown. (b) Powder XRD pattern of a powered sample of xerogel **1**. Low–medium-angle XRD patterns obtained in geometries having the X-ray beam normal (c) and parallel (d) to the surface of a xerogel film fragment.

The freeze drying of hydrogel **1** produced the xerogel **1**, which appeared as a fractured film (Figure 3a inset).

In this film a fibrous structure is observed (Figure 3a). The fibers appear locally organized in bundles, which are randomly oriented. The powder XRD pattern of xerogel **1** shows several diffraction peaks at high and medium angles (Figure 2b), indicating the presence of ordered structures in the xerogel. The periodic distances at 1.8 nm and 0.54 nm were also observed in the diffraction pattern from the parent hydrogel **1**. The XRD patterns from a film fragment of the xerogel **1**, which were obtained in geometries having the X-ray beam normal (c) and parallel (d) to the film surface (Figure 2c and Figure 2d), show diffraction effects, i.e., arcs and rings, respectively, with the same periodicities at 2.5 nm, 2.2 nm, 1.7 nm, 1.05 nm, 0.67 nm and 0.49 nm. This different distribution of the intensity of diffraction indicates that the ordered regions are randomly oriented on the film surface and preferentially oriented in the film cross section. The arcs at 2.5 nm, 2.2 nm, 1.7 nm, 1.05 nm, 0.67 nm, 0.54 nm and 0.49 nm are along the equatorial direction, while that at 0.54 nm is along the meridional direction. These low-angle periodicities could be associated with different diffraction orders of a periodic distance of 2.5 nm. This observation, in agreement with a rough estimation of the dimensions of molecule **1**, may suggest a supramolecular assembly of molecules **1** to form the fibrous structures. Moreover, XRD data and SEM observation show that in the xerogel the fibers align preferentially parallel to the film surface and may suggest that in the fiber the molecules are organized in layers (0.54 nm thick) and that they have their long axis (2.5 nm) parallel to the fiber direction. It is important to note that the low-angle periodicities observed in the xerogel were present also in the hydrogel. This indicates that the fiber structure is not generated by the freeze-drying process but that is already present in the hydrogel.

**Figure 4:**
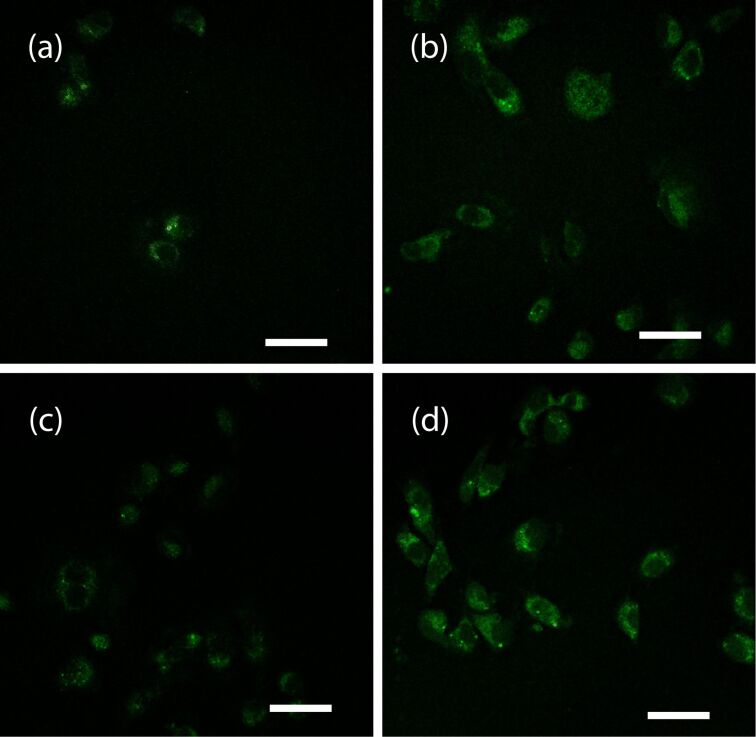
Confocal laser scanning micrographs of IGROV1 exposed to hydrogel **6** (a), to hydrogel **7** (b), to hydrogel **8** (c) and to hydrogel **9** (d). The dansyl group fluorescence is shown in green. Scale bar 50 μm.

We then collected some information on the ability of fluorescent hydrogels **6–9** to cross the cellular membrane of the cancer cells, by means of confocal microscopy (Figure 4). It is worth mentioning that gel **6** is made of **B**, while gels **7–9** are made of **A**, doped with one of the dansyl containing compounds **B–D**. IGROV-1 cells were grown on glass coverslips for 24 h before being exposed to hydrogels for 30 min. Confocal laser scanning microscopy shows that all the hydrogels internalize in IGROV-1 cells (as indicated by the green fluorescence in the cytoplasm Figure 4), but very few green spots are present in Figure 4a, suggesting that hydrogel **6** is not well tolerated by the cells.

**Figure 5:**
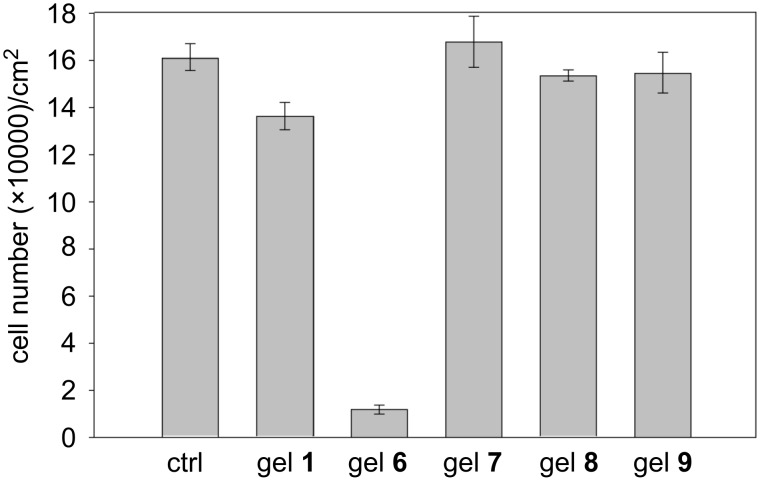
IGROV1 cell growth after 24 hour treatment with hydrogels **1, 6–9**.

To confirm these results, we evaluated the cytotoxicity of the hydrogels **1** (only **A**), compared with the fluorescent hydrogels **6** (only **B**) and **7–9** (doped **A**). IGROV-1 cell growth was evaluated after 24 h treatment with the hydrogels. The results are reported in Figure 5 and show that **B** (gel **6**) is cytotoxic, while **A** is well tolerated by the cells, as the treatment with gels **1** and **7–9** does not significantly affect the cell proliferation.

Thus, LMWHG **A** may behave as an excellent Trojan Horse as it is biologically inactive, is internalized by cells, can carry molecules of different sizes as cargo, and is a good candidate to carry drug molecules.

## Conclusion

In conclusion, we have shown the formation of a wide variety of hydrogels, that may be formed by different water/ethanol ratios and the gelator CH_2_(C_3_H_6_CO-L-Phe-D-Oxd-OH)_2_ **A**, which may also be doped with small amounts of dansyl-containing compounds, needed to show the cellular uptake into IGROV-1 cells by confocal laser scanning microscopy. These gels are readily internalized by cells and are biologically inactive. In contrast, the hydrogel **6** formed only by **B** and a water/ethanol mixture is cytotoxic. This research represents the first step in the production of a LWMHGs family that is (i) responsive to external stimuli, (ii) nontoxic and able to be internalized by cells, and (iii) easy to functionalize to be aimed toward different substrates. Thus, this may represent a new tool for the controlled release of drugs into specific cells.

## Experimental

### Synthesis and Characterization

**General:** The melting points of the compounds were determined in open capillaries and are uncorrected. High-quality infrared spectra (64 scans) were obtained at 2 cm^−1^ resolution by using a 1 mm NaCl solution cell and a Nicolet 210 FT-infrared spectrometer. All spectra were obtained from 3 mM solutions in dry CH_2_Cl_2_ at 297 K or from a 1% solid mixture with dry KBr. All compounds were dried in vacuo and all the sample preparations were performed in a nitrogen atmosphere. Routine NMR spectra were recorded with spectrometers at 400 MHz (^1^H NMR) and at 100 MHz (^13^C NMR). The measurements were carried out in CDCl_3_, in DMSO-*d*_6_ or in CD_3_OD. The proton signals were assigned by *g*COSY spectra. Chemical shifts are reported in δ values relative to the solvent peak.

**Boc-L-Phe-D-Oxd-L-Lys(Dansyl)OBn:** As described in [23], a solution of Boc-L-Lys(Dansyl)-OBn (0.22 mmol, 0.13 g) and TFA (4.06 mmol, 0.31 mL) in dry dichloromethane (10 mL) was stirred at room temperature for 4 h, then the volatiles were removed under reduced pressure and the corresponding amine salt was obtained pure in quantitative yield without further purification.

A solution of Boc-L-Phe-D-Oxd-OH (0.22 mmol, 0.09 g) and HBTU (0.22 mmol, 0.9 g) in dry acetonitrile (15 mL) was stirred under a nitrogen atmosphere for 10 min at room temperature. Then a mixture of the previously obtained amine salt (0.22 mmol) and Et_3_N (0.67 mmol, 0.24 mL) in dry acetonitrile (10 mL) was added dropwise at room temperature. The solution was stirred for 40 min under a nitrogen atmosphere, and then the acetonitrile was removed under reduced pressure and replaced with ethyl acetate. The mixture was washed with brine (1 × 15 mL), 1 N aqueous HCl (1 × 15 mL) and with 5% (w/v) aqueous NaHCO_3_ (1 × 15 mL), dried over sodium sulfate and concentrated in vacuo. The product was obtained pure after silica-gel chromatography (cyclohexane/ethyl acetate 85:15 → cyclohexane/ethyl acetate 70:30 as eluant) in 69% overall yield. Mp 177–178 °C; [α]_D_^20^ +26.9 (*c* 0.5, CHCl_3_); IR (CH_2_Cl_2_, 3 mM) ν: 3682, 3599, 3432, 3348, 1787, 1740, 1696 cm^−1^; ^1^H NMR (CDCl_3_, 400 MHz) δ 0.78–0.91 (m, 1H, CHCH_2_CH*H*), 1.15–1.85 (m, 17H, C*H*_2_C*H*HC*H*_2_CH_2_NH + OCHC*H*_3_ + *t*-Bu), 2.74–3.00 (m, 9H, CHN-C*H*H-Ph + C*H*_2_-NH-SO_2_ + N(CH_3_)_2_), 3.08–3.18 (m, 1H, CHN-CH*H*-Ph), 4.28 (d, *J* = 5.2 Hz, 1H, CHNOxd), 4.49–4.62 (m, 2H, CH(Lys) + CHO-Oxd), 5.12 (AB, *J*_1_ = 44 Hz, *J*_2_ = 12.4 Hz, 2H, CH_2_OBn), 5.03 (d, *J* = 12.4 Hz, 1H, C*H*HPh), 5.15 (d, *J* = 12.4 Hz, 1H, CH*H*Ph), 5.44 (bs, 1H, NH-Boc), 5.51 (d, *J* = 6.4 Hz, 1H, CONH), 5.58–5.70 (m, 1H, CH(Phe)), 7.15–7.35 (m, 13H, 2 × Ph + H-6*^1^* + H-3*^1^* + NH(Phe)), 7.46–7.55 (m, 2H, H-7*^1^* + H-4*^1^*), 8.21 (d, *J* = 7.6 Hz, 1H, H-2*^1^*), 8.34 (d, *J* = 8.8 Hz, 1H, H-8*^1^*), 8.53 (d, *J* = 8.8 Hz, 1H, NHSO_2_); ^13^C NMR (CDCl_3_, 100 MHz) δ 15.2, 20.8, 22.0, 28.2, 42.5, 45.3, 51.8, 54.0, 63.1, 65.8, 67.0, 74.7, 80.6, 115.2, 118.9, 123.2, 127.2, 128.2, 128.4, 128.6, 129.4, 129.5, 129.6, 129.9, 130.2, 135.0, 135.2, 135.5, 151.4, 167.1, 171.0, 174.1; Anal. calcd for C_37_H_47_N_5_O_10_S: C, 58.95; H, 6.28; N, 9.29; found: C, 59.03; H, 6.23; N, 9.24.

**CH_2_(C_3_H_6_CO-L-Phe-D-Oxd-L-Lys(Dansyl)OBn)_2_:** As described in [23], a solution of Boc-L-Phe-D-Oxd-L-Lys(Dansyl)-OBn (0.24 mmol, 0.2 g) and TFA (4.32 mmol, 0.33 mL) in dry methylene chloride (10 mL) was stirred at room temperature for 4 h, then the volatiles were removed under reduced pressure and the corresponding amine salt was obtained pure in quantitative yield without further purification.

A solution of azelaic acid (0.12 mmol, 0.02 g) and HBTU (0.26 mmol, 0.1 g) in dry acetonitrile (15 mL) was stirred under a nitrogen atmosphere for 10 min at room temperature. Then a mixture of the previously obtained amine salt (0.24 mmol) and Et_3_N (0.72 mmol, 0.1 mL) in dry acetonitrile (10 mL) was added dropwise at room temperature. The solution was stirred for 40 min under a nitrogen atmosphere, and then the acetonitrile was removed under reduced pressure and replaced with ethyl acetate. The mixture was washed with brine (1 × 15 mL), 1 N aqueous HCl (1 × 15 mL), and 5% (w/v) aqueous NaHCO_3_ (1 × 15 mL), dried over sodium sulfate and concentrated in vacuo. The product was obtained pure after silica gel chromatography (dichloromethane 100% → dichloromethane/ethyl acetate 80:20 as eluant) in 64% overall yield. Mp 125–126 °C; [α]_D_^20^ +20.3 (*c* 0.2, CHCl_3_); IR (CH_2_Cl_2_, 3 mM) ν: 3686, 3607, 1791, 1743, 1680 cm^−1^; ^1^H NMR (DMSO-*d*_6_, 400 MHz) δ 0.80–1.03 (m, 10H, COCH_2_CH_2_(C*H*_2_)_3_ + 2 × CHCH_2_C*H*_2_), 1.05–1.31 (m, 8H, 2 × COCH_2_C*H*_2_ + 2 × C*H*_2_CH_2_NH), 1.35 (d, *J* = 7.6 Hz, 6H, 2 × OCHC*H*_3_), 1.44–1.61 (m, 4H, 2 × CHC*H*_2_), 1.85–2.03 (m, 4H, 2 × COC*H*_2_), 2.62–2.73 (m, 8H, 2 × CHN-C*H*H-Ph + 2 × C*H*_2_-NH-SO_2_), 2.79 (s, 12H, 2 × N(CH_3_)_2_), 3.03–3.12 (m, 2H, 2 × CHN-CH*H*-Ph), 4.14–4.23 (m, 2H, 2 × CH(Lys), 4.39–4.48 (m, 4H, 2 × CHNOxd + 2 × CHO-Oxd), 5.02–5.10 (m, 4H, 2 × CH_2_OBn), 5.69–5.80 (m, 2H, 2 × CH(Phe), 7.15–7.35 (m, 20H, 4 × Ph), 7.53–7.62 (m, 4H, 2 × H-6*^1^* + 2 × H-3*^1^*), 7.82–7.88 (m, 2H, 2 × H-7*^1^*), 8.07 (d, *J* = 8.0 Hz, 2H, 2 × H-4*^1^*), 8.18 (d, *J* = 8.8 Hz, 2H, 2 × NH(Phe)), 8.27 (d, *J* = 8.8 Hz, 2H, 2 × H-2*^1^*), 8.44 (d, *J* = 8.8 Hz, 2H, 2 × H-8*^1^*), 8.71 (d, *J* = 8.8 Hz, 2H, 2 × NHSO_2_); ^13^C NMR (DMSO-*d*_6_, 100 MHz) δ 25.4, 27.2, 30.2, 33.4, 33.7, 35.5, 40.2, 43.4, 47.3, 50.2, 57.0, 57.5, 60.0, 67.0, 71.2, 80.1, 120.3, 128.7, 131.7, 133.0, 133.2, 133.6, 134.2, 134.3, 140.9, 141.1, 142.3, 156.5, 157.3, 172.8, 176.4, 177.2, 177.4, 179.8; Anal. calcd for C_87_H_102_N_10_O_18_S_2_: C, 63.72; H, 6.27; N, 8.54; found: C, 63.76; H, 6.30; N, 8.55.

**CH_2_(C_3_H_6_CO-L-Phe-D-Oxd-L-Lys(Dansyl)OH)_2 _(B):** As described in [23], compound CH_2_[C_3_H_6_CO-L-Phe-D-Oxd-L-Lys(Dansyl)-OBn]_2_ (0.06 mmol, 0.1 g) was dissolved in MeOH (20 mL) under nitrogen. C/Pd (20 mg) was added under nitrogen. A vacuum was created inside the flask using the vacuum line. The flask was then filled with hydrogen from a balloon (1 atm). The solution was stirred for 5 hours under a hydrogen atmosphere. The product was obtained pure in quantitative yield after filtration through a celite pad using MeOH and concentration in vacuo. Mp 125–126 °C; [α]_D_^20^ + 22.7 (*c* 0.3, MeOH); ^1^H NMR (CD_3_OD, 400 MHz) δ 0.86–1.66 (m, 28H, COCH_2_(C*H*_2_)_5_ + 2 × CH(CH_2_)_3_CH_2_NH + 2 × OCHC*H*_3_), 1.90–2.08 (m, 4H, 2 × COC*H*_2_), 2.62–2.81 (m, 18H, 2 × CHN-C*H*H-Ph + 2 × C*H*_2_-NH-SO_2_ + 2 × N(CH_3_)_2_), 3.06–3.15 (m, 2H, 2 × CHN-CH*H*-Ph), 4.08–4.20 (m, 2H, 2 × CH(Lys)), 4.27 (d, *J* = 5.2 Hz, 2H, 2 × CHNOxd), 4.45–4.55 (m, 2H, 2 × CHO-Oxd), 5.72–5.82 (m, 2H, 2 × CH(Phe)), 7.04–7.26 (m, 14H, 2 × Ph + 2 × H-6*^1^* + 2 × H-3*^1^*), 7.42–7.52 (m, 4H, 2 × H-7*^1^* + 2 × H-4*^1^*), 8.04–8.12 (m, 2H, 2 × NH(Phe)), 8.06–8.27 (m, 4H, 2 × H-2*^1^* + 2 × H-8*^1^*), 8.41–8.49 (m, 2H, 2 × NHSO_2_); ^13^C NMR (CD_3_OD, 100 MHz) δ 21.8, 24.7, 27.6, 30.6, 30.9, 32.5, 37.4, 39.3, 39.7, 44.5, 46.7, 55.4, 65.5, 77.3, 117.3, 121.4, 125.2, 128.9, 130.0, 130.3, 131.0, 131.3, 131.8, 137.9, 138.8, 154.0, 154.5, 171.1, 174.8, 177.0; Anal. calcd for C_73_H_90_N_10_O_18_S_2_: C, 60.07; H, 6.21; N, 9.60; found: C, 60.03; H, 6.19; N, 9.57.

**Dansylamide (C)** may be purchased by Sigma-Aldrich.

**Dansylpyrrolidine** (**D):** A solution of dansyl chloride (1.11 mmol, 0.3 g) and pyrrolidine (0.55 mmol, 0.5 mL) in dry dichloromethane (20 mL) was stirred under a nitrogen atmosphere for 2 hours at room temperature. The mixture was washed with brine (1 × 30 mL) and 1 N aqueous HCl (2 × 30 mL), and the organic layer was dried over sodium sulfate and concentrated in vacuo. The product was obtained pure without further purification as a green solid in 86% overall yield. Mp 102 °C; ^1^H NMR (CDCl_3_, 400 MHz) δ 1.75–1.81 (m, 4H, 2 × NCH_2_CH_2_), 2.83 (s, 6H, N(CH_3_)_2_), 3.27–3.33 (m, 4H, 2 × NCH_2_CH_2_), 7.13 (d, *J* = 8.0 Hz, 1H, H-61), 7.45–7.52 (m, 2H, H-31 + H-71), 8.17 (d, *J* = 8.0 Hz, 1H, H-41), 8.44 (d, *J* = 8.4 Hz, 1H, H-21), 8.52 (d, *J* = 7.2 Hz, 1H, H-81); ^13^C NMR (CDCl_3_, 100 MHz) δ 25.4, 45.3, 47.4, 115.0, 129.7, 123.1, 127.8, 129.5, 129.9, 130.1, 130.5, 134.2, 151.5.

### Conditions for hydrogel formation

**Hydrogel 1:** As described in [22], compound **A** (3.7 mg, 5 μmol) was dissolved in ethanol (50 μL) in a test tube (8 mm wide) and shaken for a few minutes by hand. Then, water (450 μL) was added, and the tube was sonicated for fifteen minutes at room temperature and then left to stand for 16 hours for the gel formation. The xerogel was obtained, after solvent evaporation at room temperature.

**Hydrogel 2:** As described in [22], compound **A** (3.7 mg, 5 μmol) was dissolved in ethanol (250 μL) in a test tube (8 mm wide) and shaken for a few minutes by hand. Then, water (250 μL) was added, and the tube was sonicated for fifteen minutes at room temperature and then left to stand for 16 hours for the gel formation.

**Hydrogel 3:** As described in [22], compound **B** (3.7 mg, 2.5 μmol) was dissolved in ethanol (50 μL) in a test tube (8 mm wide) and shaken for a few minutes by hand. Then, water (450 μL) was added, and the tube was sonicated for fifteen minutes at room temperature and then left to stand for 16 hours. Unfortunately, no gel formed.

**Hydrogel 4:** As described in [22], compound **B** (3.7 mg, 2.5 μmol) was dissolved in ethanol (250 μL) in a test tube (8 mm wide) and shaken for a few minutes by hand. Then, water (250 μL) was added, and the tube was sonicated for fifteen minutes at room temperature and then let stand for 16 hours. Unfortunately, no gel formed.

**Hydrogel 5:** As described in [22], compound **B** (7.3 mg, 5 μmol) was dissolved in ethanol (50 μL) in a test tube (8 mm wide) and shaken for a few minutes by hand. Then, water (450 μL) was added, and the tube was sonicated for fifteen minutes at room temperature and then left to stand for 16 hours. Unfortunately, no gel formed.

**Hydrogel 6:** As described in [22], compound **B** (7.3 mg, 5 μmol) was dissolved in ethanol (250 μL) in a test tube (8 mm wide) and shaken for a few minutes by hand. Then, water (250 μL) was added, and the tube was sonicated for fifteen minutes at room temperature and then left to stand for 16 hours for the gel formation.

**Hydrogel 7:** As described in [22], compound **A** (3.7 mg, 5 μmol) and compound **B** (0.4 mg, 0.025 μmol) were dissolved in ethanol (50 μL) in a test tube (8 mm wide) and shaken for a few minutes by hand. Then, water (450 μL) was added, and the tube was sonicated for fifteen minutes at room temperature, and then left to stand for 16 hours for the gel formation.

**Hydrogel 8:** As described in [22], compound **A** (3.7 mg, 5 μmol) and compound **C** (0.4 mg, 0.16 μmol) were dissolved in ethanol (50 μL) in a test tube (8 mm wide) and shaken for a few minutes by hand. Then, water (450 μL) was added, and the tube was sonicated for fifteen minutes at room temperature and then left to stand for 16 hours for the gel formation.

**Hydrogel 9:** As described in [22], compound **A** (3.7 mg, 5 μmol) and compound **D** (0.4 mg, 0.13 μmol) were dissolved in ethanol (50 μL) in a test tube (8 mm wide) and shaken for a few minutes by hand. Then, water (450 μL) was added, and the tube was sonicated for fifteen minutes at room temperature, then left to stand for 16 hours for the gel formation.

### Description of the analysis conditions

**Microscopy:** As described in [22], hydrogels and xerogels were systematically observed by optical microscopy (OM) and scanning electron microscopy (SEM), respectively. The OM images were collected by using an optical microscope (Leica) equipped with a CCD camera. SEM images of xerogels were collected after gluing the samples on a carbon tape and observed directly in a Phenom microscope (FEI) without any coating procedure.

**X-ray powder diffraction analysis:** As described in [22], X-ray powder diffraction patterns were collected by using a PanAnalytical X’Pert Pro equipped with an X’Celerator detector powder diffractometer using Cu Kα radiation generated at 40 kV and 40 mA. The diffraction patterns were collected within the 2Θ range from 5° to 35° with a step size (Δ2Θ) of 0.02° and a counting time of 1200 s.

**Atomic absorption spectroscopy analysis:** Elemental platinum concentrations in medium solutions were measured with a Perkin-Elmer Mod. AAnalyst 100 Absorption Spectrometer (Perkin-Elmer Co., Norwalk, CT) equipped with a deuterium background corrector, Autosampler AS-72, graphite furnace Perkin-Elmer Mod. HGA-800. A Pt Lumina (Perkin-Elmer) hollow-cathode lamp was used. Ten microliters of sample was injected and the furnace was heated slowly to 2500 °C. The absorbance of atomized platinum was measured at 265.7 nm.

**Cell culture and treatments:** The ovarian cancer cell line IGROV-1 was kindly provided by Prof. Colnaghi Istituto Nazionale Tumori Milan, Italy. The cells were maintained in RPMI 1640 medium (Labtek Eurobio, Milan, Italy), supplemented with 10% FCS (Euroclone, Milan, Italy) and 2 mM L-glutamine (Sigma-Aldrich) at 37 °C and 5% CO_2_. Cells were detached with 0.11% trypsin/0.02% EDTA (Sigma-Aldrich), seeded at 1 × 10^4^ cells/cm^2^ in multiwell plates (Orange Scientific, Braine-l’Alleud, Belgium), and allowed to grow for one day before being exposed to 2.9 µM hydrogel. The effects on the proliferation were studied after 24 h of treatment. Cell viability was determined by propidium iodide dye exclusion.

**Confocal laser scanning microscopy:** For confocal microscopy, IGROV 1 were grown on glass coverslips for 24 h before being exposed to hydrogels **6**, **7**, **8** and **9** for 30 min. Cells were washed in PBS and then fixed with 3% paraformaldehyde and washed with 0.1 M glycine in PBS and 1% BSA in PBS. Preparations were embedded in Mowiol, and the images were acquired by using sequential laser excitations at 488 nm. The images were collected by using a Nikon C1s confocal laser scanning microscope, equipped with a Nikon PlanApo 60, 1.4-NA oil immersion lens.

**Statistical analysis:** All values are expressed as mean ± SE. Student two-tailed paired *t*-test was used for all statistical analyses.
